# The Obesity-Related Gut Bacterial and Viral Dysbiosis Can Impact the Risk of Colon Cancer Development

**DOI:** 10.3390/microorganisms8030431

**Published:** 2020-03-19

**Authors:** Giuseppina Campisciano, Nicolò de Manzini, Serena Delbue, Carolina Cason, Davide Cosola, Giuseppe Basile, Pasquale Ferrante, Manola Comar, Silvia Palmisano

**Affiliations:** 1 SSD of Advanced Microbiology Diagnosis and Translational Research, Institute for Maternal and Child Health-IRCCS “Burlo Garofolo”, Via dell’Istria 65/1, 34137 Trieste, Italy; giusi.campisciano@burlo.trieste.it; 2 General Surgery Clinic, Azienda Sanitaria Universitaria Giuliano Isontina (ASU GI), Strada di Fiume 447, 34149 Trieste, Italy; ndemanzini@units.it (N.d.M.); d.cosola84@gmail.com (D.C.); spalmisano@units.it (S.P.); 3 Department of Medical, Surgical and Health Sciences, Azienda Sanitaria Universitaria Giuliano Isontina (ASU GI), Strada di Fiume 447, 34149 Trieste, Italy; 4 Department of Biomedical, Surgical and Dental Sciences, Laboratory of Translational Research, Via Carlo Pascal 36, 20133 Milano, Italy; serena.delbue@unimi.it (S.D.); pasquale.ferrante@unimi.it (P.F.); 5 Clinical Institute San Siro, Via Monreale 18, 20148 Milano, Italy; giuseppe.basile@unife.it

**Keywords:** microbiome, cancer pathogenesis, dysbiotic microbiota, obesity, gut microbiota

## Abstract

An incorrect food regimen from childhood is suggested to negatively impact the gut microbiome composition leading to obesity and perhaps to colon rectal cancer (CRC) in adults. In this study, we show that the obesity and cancer gut microbiota share a characteristic microbial profile with a high colonization by mucin degraders species, such as *Hafnia alvei* and *Akkermansia muciniphila*. In addition, the species *Clostridium* *bolteae,* a bacterium associated with insulin resistance, dyslipidemia, and inflammation, has been associated with the presence of oncogenic Human Polyomaviruses (HPyVs). Merkel cell Polyomavirus (MCPyV) and BK Polyomavirus (BKPyV) were the most frequently oncogenic viruses recovered in the gut of both obese and tumor patients. Considering the high seroprevalence of HPyVs in childhood, their association with specific bacterial species deserve to be further investigated. Data from the present study highlight the presence of a similar microbiome pattern in CRC and obese subjects, suggesting that obese microbiome may represent an opportunity for tumorigenic/driver bacteria and viruses to trigger cell transformation.

## 1. Introduction

Colorectal cancer (CRC) is a major public health concern, as it is one of the leading causes of cancer deaths in the Western world [1]. CRC requires decades to clinically manifest, starting from the initial transformed intestinal crypt cells [2]. Thus, this means that some risk factors could be traced back to the childhood and early adulthood. Especially the increase in the CRC incidence is considered a consequence of the modern life-style and is associated with calorically excessive high-fat/low-fiber diet, consumption of refined products, lack of physical activity, and obesity [3,4]. This association seems to be stronger when childhood overweight persists into early adulthood than when the overweight disappears before early adulthood or develop after childhood [5].

An unhealthy diet impacts gut microbial composition, triggering oncogenic transformation, conducive to the alteration of metabolic pathways, including insulin resistance, hyperinsulinemia, unbalanced level of growth factors, adipocytokines and steroid hormones [6]. Therefore, the time of exposure to these metabolic changes, especially if present since childhood, may increase the chances for the development of CRC in adulthood.

As of yet, a strong universal obesity-related microbial profile linked to CRC development has not been identified, due to the interpersonal variability driving CRC, including genetic factors, behavioral style, and diet. Nonetheless, the CRC ‘driver-passenger’ model suggests that symbiotic ‘driver’ bacteria initiate tissue malignant transformation through cell DNA damage and that colorectal tumorigenesis is successively mediated by alterations of the intestinal microenvironment, conducive to the proliferation of “passengers” opportunistic pathogens, such as *Fusobacterium* spp., *Streptococcus bovis* and *Roseburia* spp. [7]. Among “passengers” players, Human Polyoma Viruses (HPyVs) can also be mentioned. Interestingly, despite their known transformative abilities, HPyVs infections are often asymptomatic, with an age profile indicating high frequency of early-age infections and lifelong persistence [8,9,10].

To note, gut bacterial and viral dysbiosis in both CRC and obese patients are strongly responsible for altered host immune response, leading to a chronic inflammatory status [11].

The importance of resident bacteria in regulating the gut homeostasis has been reported for a group of gut colonizer microorganisms. Specifically, the alteration of quantitative relationship between bacterial composition in the intestinal lumen and mucosa has been described in patients with CRC, characterized by a decreased rate of colonization of *Bifidobacterium*, *Faecalibacterium,* and *Blautia* and the predominance of *Porphyromonas* and *Mogibacterium* species [12]. Moreover, the presence of “not gut” bacteria including *Fusobacterium nucleatum* and *Fusobacterium necrophorum*, commonly inhabiting the oral cavity, and occasionally causing periodontal and gingival infections, have been found repeatedly associated with CRC [13,14].

At the same time, metagenomics analysis of gut of obese subjects showed an inverse ratio of *Bacteroidetes* to *Firmicutes* family, with high level of *Lactobacillus* species and with a relatively low level of *Bacteroides* [15].

Considering the dramatic increase of worldwide childhood obesity [16], understanding whether bacterial dysbiosis due to prolonged unhealthy diet, leading to obesity, plays a role in driving a more susceptible gut environment for CRC would represent an urgency in order to draw targeted preventive diet recommendations, starting from the early stage of life. In this regard, we wanted to assess whether the gut bacterial composition of obese patients, who were on an unhealthy diet from childhood, resembles that of patients affected with CRC.

## 2. Materials and Methods

### 2.1. Case Study

Stool samples were collected before surgery, as requested by the clinical practice, from 53 patients with CRC, eligible for surgery (Tumor Group, age 74 ± 11), and from 25 obese subjects, eligible for bariatric surgery (Obese Group, age 44,5 ± 9,5), present at the Azienda Sanitaria Universitaria Giuliano Isontina (ASU GI), Trieste, Italy. Additionally, 27 healthy normal weight volunteer subjects (Control Group, age 44,2 ± 9,3) were enrolled in the study. Clinical procedures have been completed in accordance with Good Clinical Practices and the ethical standards according to the Declaration of Helsinki.

Inclusion criteria for obese and control patients were in line with international bariatric guidelines [17]. Inclusion criteria for patients with CRC were primarily based on patients’ willingness to participate in the study. Informed consent was obtained from all participants and the privacy rights of human subjects were observed. Exclusion criteria were familiarity for colorectal cancer, the presence of inflammatory bowel diseases and administration of antibiotic/probiotic therapy within 1 month before the study enrollment.

### 2.2. 16S rRNA Gene Library Preparation Targeted Sequencing

The NucliSENS^®^ easyMAG system (bioMèrieux, Marcy l’Etoile, France) was used for nucleic acid extraction. In brief, 100 mg of fecal sample were incubated with 1.8 mL of Lysis Buffer easyMAG and 50 µg/mL of proteinase K (Euroclone, Trieste, Italy), vortexed until the fecal sample was thoroughly homogenized and incubated at 56 °C for 3 h with continuous shaking. The samples were centrifuged for 15 min at 13,000 x *g* and 500 µL of the supernatant were used for the extraction, with an elution volume of 50 μL.

An EVAGREEN (EvaGreen^®^ dye, Fisher Molecular Biology, Waltham, MA, USA) real-time polymerase chain reaction (PCR) using the 27FYM degenerated primer (5′-AGR GTT YGA TYM TGG CTC AG-3′) and the U534R primer (targeting the V1-V3 region of 500 bp length) was performed. A nested PCR was performed with the primers B338F_P1-Ion-adaptor (B338F 5′-ACTCCTACGGGAGGCAGC-3′) and U534R_A-Ion-adaptor_IonXpress-barcode (U534R 5′-ATTACCGCGGCTGCTGG-3′), in order to amplify the V3-region template of 200 bases for sequencing analysis. Negative controls including no template were processed with clinical samples. The Kapa 2G HiFi Hotstart ready mix 2X (Kapa Biosystems, Wilmington, Massachusetts, USA) was used for the amplifications and 400 ng/µL BSA were added to the reaction mix. The temperature cycling was 5 min at 95 °C, 30 s at 95 °C, 30 s at 59 °C (for V1-V3 region)/57 °C (for V3 region), 45 s at 72 °C and a final elongation step at 72 °C for 10 min.

Qubit^®^ 2.0 Fluorometer (Invitrogen, Carlsbad, CA, USA) was used for the quantification of the amount of dsDNA. An equal amount of each PCR reaction was pooled into a single batch and the pooled-library diluted to a concentration of 100 pM.

Template preparation was performed using the Ion PGM Hi-Q View kit on Ion OneTouch™ 2 System (Life Technologies, Gran Island, NY, USA) and sequenced using the Ion PGM Hi-Q View sequencing kit (Life Technologies, Gran Island, NY, USA) by the Ion PGM™ System technology.

### 2.3. Bioinformatics Analysis

QIIME 1.9.1 software (Quantitative Insights into Microbial Ecology) was used to process the sequence data [18]. High quality (Q > 20) sequences with a minimum length of 150 bp were retained for the analyses. Operational taxonomic units (OTUs) were assigned at 97% similarity and clustered against the Human Intestinal Database [19] using open-reference OTU picking [20] with a uclust clustering tool [21]. Before further analysis, singleton OTUs and samples with low sequencing depth were removed (less than 10,000 reads) and a cumulative sum scaling was applied.

### 2.4. Detection of the HPyV Genomes

Q-PCR reactions specific for the six HPyV analyzed (JCPyV, BKPyV, Merkel cell PyV–MCPyV-, HPyV-6, HPyV-7, and HPyV-9) were performed, using 1X Taqman Universal PCR Master Mix (Applied Biosystems, Foster City, CA, USA), 0,4 μM primer Forward, 0,4 to 0,9 μM primer Reverse, 0,2 μM probe, and 250 ng of extracted nucleic acid, in a final volume of 25 μL [22]. Thermal cycling was performed as follows: denaturation step at 95 °C for 10 min, followed by 40 cycles of 95 °C for 15 s and 60 °C for 1 min, at the end of which the fluorescence was acquired. A 10-fold dilution series of plasmids containing the entire VP1 gene of BKPyV, MCPyV, HPyV-6, HPyV-7, and HPyV-9 and the entire LT of JCPyV (dilution range: 108–10 copies/μL) was used to construct the standard curves. The limit of detection of each assay was 10 copies/μL. To determine the quality and percentage of infected cells, a concomitant Q-PCR assay targeting the β-globin gene was performed on the same samples using the primer set and thermal cycles previously published [23].

Viral loads were expressed as copies/μg of DNA extracted. Negative and positive controls were included in each run. Each sample, standard and control, was tested in triplicate. The results were analyzed by the absolute quantification method and reported as copies/mL and percentage of infected cells, calculated as follows: ([viral copies]/[beta-globin copies/2]) × 100.

### 2.5. Statistical Analysis

Differences in microbial community composition were investigated using QIIME 1.9.1. Chao1, Observed species, and Shannon metrics were used to assess alpha diversity (within-sample diversity), while the LEfSe test (Linear discriminant analysis Effect Size) for beta diversity (between sample diversity comparison). To test whether any individual bacterium specifically correlated with a clinical metadata (sex, age, body mass index (BMI), diabetes, smoke, alcohol, HPyVs), the observation_metadata_correlation.py script, using bootstrapping and Pearson correlation, was used. To test microbial differences between positive and negative samples for HPyVs, a non-parametric T Test was used. All the statistical analyses were performed after rarefying the otu_table.biom (depth 10,000 reads/sample) and applying a cumulative sum scaling.

## 3. Results

We performed the sequencing of the V3 region of the16S rRNA gene from 107 biological samples including 53 stool samples from CRC patients (Tumor group), 27 stool samples from obese patients (Obese group), and 27 healthy normal weight patients (Control group). Table 1 shows the patients’ demographics. The two negative controls analyzed did not produce any sequencing output after the quality filtering. The sequencing of the remaining samples produced a total of 5206,621 reads (Q score > 20, range 5519–160,737) and a total number of observed OTUs of 11,815 (the reads were clustered into 200 ± 50 OTUs per sample).

### 3.1. Alpha Diversity

Alpha diversity was quantified by Chao1 diversity index, by the total number of observed species, and by Shannon index. Table 2 shows the alpha diversity measurements for Tumor, Obese, and Control groups. Statistical testing showed no difference for Chao1 richness estimator and the observed species (*p* value > 0.05). Nevertheless, a decrease trend of alpha diversity was observed in the Tumor group for Chao1 and Observed species metrics.

Shannon, which relates both OTU richness and evenness, did not reveal a significant change. However, the Tumor group showed a slight decrease trend towards the value of the Obese group (Table 2).

### 3.2. Specific OTUs

The observation_metadata_correlation.py script did not reveal any significant association of specific bacteria with the clinical metadata, such as diabetes, alcohol, smoke, BMI, sex, and HPyV. Thus, excluding cofounding factors in the observed differences between clinical groups.

Next, the LEfSe test was used in order to identify significantly imbalanced biomarkers, which showed the strongest effects for group differentiation. Analysis at the phylum level showed that *Verrucomicrobia* (*p* value 0.004, LDA score 4.8) were significantly higher in the Tumor group (Table 3).

At the species level, the increased amount of *Proteobacteria* and *Verrucomicrobia* in the Tumor group were dependent mainly on the increase of *Hafnia alvei* (7% ± 11%), which had the highest LDA score, and *Akkermansia muciniphila* (1% ± 2%), respectively. Among *Bacteroidetes*, there was a different distribution of species among groups, with *Alistipes senegalensis* higher in the Tumor group (1.5% ± 3%) while *Barnesiella intestinihomins* was higher in the Control group (0.11% ± 0.18%). Among *Firmicutes*, several species were identified as biomarkers by LEfSe test. Among these, *Blautia wexlerae, Eubacterium rectale, Lactobacillus rogosae,* and *Ruminococcus faecis* showed a similar relative abundance between Tumor and Obese groups (Table 4).

### 3.3. Microbiome Composition of HPyV-Positive Samples

Overall, HPyV genomes were detected in seven stool samples. Of the six searched HPyVs, only BKPyV and MCPyV were detected. Precisely, BKPyV was identified in 2/53 (3.8%) of stool samples from the Tumor group, while MCPyV was equally distributed between samples from Tumor (4/53; 7.6%) and Obese groups (2/27; 7.4%). One out of 27 (3.7%) samples from the Control group showed MCPyV infection (Table 5).

Comparing the microbiome profile of the HPyV positive with the HPyV negative samples by a non-parametric T test, using the rarefied biom table (10,000 reads/sample), some differences were observed. Precisely, a significant (*p* value = 0.014) higher relative abundance of *Clostridium bolteae* was observed in HPyVs positive samples comparing with negative samples (0.27% ± 0.63% vs. 0.07% ± 0.17, respectively).

## 4. Discussion

Data from the present study suggest a similar composition of the gut microbiome from CRC and obese patients. It is plausible to assert that dysbiosis usually observed in obese people [24] represents an opportunity for “tumorigenic/driver bacteria” to proliferate, and to elicit a low-grade persistent mucosal inflammation which turns into cell damage. Thus, as long as the obesity condition persists, it is more likely for an oncogenic transformation to take place, especially when the unhealthy diet starts from childhood.

We compared the gut microbiota of the CRC group with the gut dysbiotic microbiota of obese subjects, with a known history of unhealthy diet and obesity since childhood, and with the gut eubiotic microbiota of normal-weight subjects. Our aim was to highlight overlapping microbial signatures that can lead to an increased understanding of the bacterial activity in the pathogenesis of CRC. These shared microbial signatures can be of great impact to define a tailored preventive dysbiosis correction since the early stage of life and especially in childhood obesity causing long-term consequences on gut microbial composition.

The alpha diversity of the gut microbiome from the three groups did not significantly differ. This is plausible considering that in the gut there is always an equilibrium in which one phylum increases at the expense of another one, without affecting the total number of microorganisms. Indeed, the Tumor group showed only a slight decrease of bacterial diversity, but, at the same time, showing a peculiar bacterial composition, suggestive of a role for microorganism in CRC. The CRC gut microbiota showed higher abundance of *Proteobacteria* and *Verrucomicrobia*, phyla usually present at low amount with respect to *Firmicutes* and *Bacteroidetes* in the gut [25,26]. The same increase trend was observed in the Obese group, suggesting a similar dysbiotic gut picture.

Most notably, this overlapping was confirmed at the species level. Within the two *Proteobacteria* and *Verrucomicrobia* phyla, we observed the increase of *Hafnia alvei* (*Proteobacteria*) and *Akkermansia muciniphila* (*Verrucomicrobia*) in the Tumor and Obese groups. These bacteria are both mucin degraders and their increase is likely a consequence of the overexpression of the two types of mucins MUC1 and MUC5AC observed in patients affected with CRC [27,28,29,30]. These data suggest a strict interplay between genic expression of the host and the bacterial response, highlighting how an altered host milieu can affect the microbial signature that, in turn, leads to inflammation and tissue damage.

When considering the presence of possible risks factors, such as HPyVs infections, the presence of these viruses correlated with a higher amount of *Clostridium bolteae* (0.27% ± 0.63% in HPyVs positive samples vs. 0.07% ± 0.17% in negative samples). *C. bolteae* has been associated with insulin resistance, dyslipidemia and inflammation [31], which are typical aspects of obese people. Thus, in this case, the driver-passenger model can comprise the interaction between a bacterial species and HPyVs, with the inflammation-associated bacterium being present decades before CRC onset, in a dysbiotic gut environment, and the HPyV infection being an adjunctive risk factor. Considering the common and often asymptomatic prevalence of HPyVs in childhood, this interaction needs to be further investigated.

## 5. Conclusions

In conclusion, identifying similarities between obesity-associated gut microbiota and CRC-associated gut microbiota holds great promise to identify key microbiota members to target in order to restore a healthy environment and to suggest preventive diet recommendation from the early stage of life in order to reduce the risk of CRC development in adulthood.

## Figures and Tables

**Table 1:** Patients’ demographics. Abbreviations: m = mean; sd = standard deviation; yrs = years; T = tumor; O = Obese; C = Control; T2DM = type 2 diabetes mellitus.

	Tumor	Obese	Control	Fisher’s Exact Test *p* Value
Age (m ± sd)	74 ± 11 yrs	44 ± 9 yrs	44 ± 9 yrs	*p* < 0.001: T vs. O/C
Women	21	21	20	*p* < 0.001: T vs. O/C
Men	32	4	7	
BMI (m ± sd)	26 ± 6 kg/m^2^	37 ± 7 kg/m^2^	22 ± 3 kg/m^2^	*p* < 0.001: O vs. T/C; *p* < 0.05: T vs. C
Smoke	13/53 (24%)	9/25 (36%)	3/27 (11%)	-
Alcohol	8/53 (15%)	0	0	*p* < 0.05: T vs. O/C
T2DM	17/53 (32%)	1/25 (4%)	0	*p* < 0.05: C vs. O/T

**Table 2:** Alpha diversity. Bacterial diversity values are given as mean ± standard deviation at a rarefaction depth of 10,000 sequences per sample. Alpha diversity was compared between pairwise comparisons by means of a non-parametric t-test using the compare_alpha_diversity.py script of QIIME. None significant variation was observed. Abbreviations: T = tumor; O = obese; C = Control.

Metric	Tumor	Obese	Control	*p* Value T vs. O	*p* Value T vs. C	*p* Value O vs. C
Chao1	559 ± 187	662 ± 201	642 ± 248	0.1	0.3	1
Observed species	289 ± 75	313 ± 72	329 ± 113	0.6	0.2	1
Shannon	4.9 ± 0.6	4.7 ± 0.5	5 ± 0.8	0.3	1	0.3

**Table 3:** Specific phyla. Linear discriminative analysis (LDA) effect size (LEfSe) test showing the phyla associated with the three groups (Tumor, Obese, Control). Positive values of LDA scores (log 10) are indicative for enriched taxa in a given group. Significant *p* values are highlighted in bold. Abbreviations: sd = standard deviation; FDR = false discovery rate.

Bacterial Phyla	Tumor		Obese		Control		*p* Value FDR	
	Mean	sd	Mean	sd	Mean	sd		LDA Score
*Actinobacteria*	1.29%	1.94%	3.42%	7.17%	2.65%	4.90%	0.38	5.1
*Bacteroidetes*	53.08%	16.11%	59.33%	21.79%	56.41%	18.82%	0.33	5.5
*Firmicutes*	32.88%	13.29%	30.14%	14.91%	35.75%	16.68%	0.41	5.4
*Fusobacteria*	0.33%	1.16%	0.33%	1.17%	0.01%	0.06%	0.22	4.1
*Lentisphaerae*	0.06%	0.16%	0.01%	0.03%	0.03%	0.07%	0.38	2.8
*Proteobacteria*	10.83%	11.96%	6.08%	7.70%	4.74%	5.57%	0.11	5.5
*Synergistetes*	0.29%	0.89%	0.14%	0.71%	0.10%	0.44%	0.11	4.0
*Verrucomicrobia*	1.10%	2.13%	0.07%	0.19%	0.03%	0.06%	**0.004**	**4.8**

**Table 4:** Specific species. Linear discriminative analysis (LDA) effect size (LEfSe) test showing the species associated with the three groups (Tumor, Obese, Control). Positive values of LDA scores (log 10) are indicative for enriched taxa in a given group. Significant *p* values are highlighted in bold. Abbreviations: m = mean; sd = standard deviation; FDR = false discovery rate; *B = Bacteroidetes; F = Firmicutes; P = Proteobacteria; V = Verrucomicrobia*.

		Tumor		Obese		Control		*p* Value FDR	
Phylum	Species	M	sd	m	sd	m	sd		LDA Score
*B*	*Alistipes putredinis*	3.03%	3.02%	0.88%	1.41%	3.02%	2.81%	**0.03**	5.0
	*Alistipes senegalensis*	1.54%	3.11%	0.41%	0.92%	0.52%	0.87%	**0.02**	4.8
	*Barnesiella intestinihominis*	0.05%	0.11%	0.02%	0.04%	0.11%	0.18%	**0.03**	2.9
*F*	*Blautia wexlerae*	0.25%	1.38%	0.46%	1.10%	0.84%	3.01%	**0.02**	4.5
	*Christensenella minuta*	0.20%	0.64%	0.00%	0.00%	0.11%	0.27%	**0.03**	4.0
	*Clostridium bartlettii*	0.32%	0.79%	1.90%	4.29%	0.29%	0.74%	**0.05**	4.9
	*Clostridium bolteae*	0.01%	0.01%	0.03%	0.08%	0.01%	0.02%	**0.00**	4.6
	*Clostridium clariflavum*	0.10%	0.22%	0.00%	0.01%	0.22%	0.40%	**0.03**	4.1
	*Eubacterium rectale*	0.03%	0.05%	0.10%	0.15%	0.35%	0.57%	**0.02**	4.2
	*Lactobacillus rogosae*	0.10%	0.15%	0.11%	0.19%	0.43%	0.54%	**0.02**	4.3
	*Papillibacter cinnamivorans*	0.16%	0.32%	0.01%	0.02%	0.38%	0.96%	**0.03**	4.3
	*Ruminococcus faecis*	0.12%	0.42%	0.12%	0.58%	0.08%	0.17%	**0.03**	3.7
*P*	*Hafnia alvei*	7.81%	11.15%	1.83%	6.82%	0.78%	1.49%	**0.00**	5.6
	*Parasutterella excrementihominis*	0.13%	0.41%	1.21%	2.90%	1.19%	2.76%	**0.02**	4.7
	*Yokenella regensburgei*	0.13%	0.50%	0.02%	0.07%	0.00%	0.00%	**0.03**	3.8
*V*	*Akkermansia muciniphila*	1.10%	2.13%	0.07%	0.19%	0.03%	0.06%	**0.01**	4.8

**Table 5:** HPyVs prevalence among patients.

Virus	Tumor (53)	Mean Viral Load (Copies/mL)	Control (27)	Mean Viral Load (Copies/mL)	Obese (27)	Mean Viral Load (Copies/mL)
BKPyV+/tot (%)	2/53 (3.8%)	6 × 10^3^	0/27		0/27	
MCPyV+/tot (%)	4/53 (7.6%)	4.2 × 10^5^	1/27 (3.7%)	8 x10^4^	2/27 (7.4%)	3.3 × 10^4^
HPyV+/tot (%)	6/53 (9.4%)	2.1 × 10^5^	1/27 (3.7%)	8 x10^4^	2/27 (7.4%)	3.3 × 10^4^
